# Clinical and molecular characteristics of extramedullary acute myeloid leukemias

**DOI:** 10.1038/s41375-024-02337-0

**Published:** 2024-07-18

**Authors:** Tariq Kewan, Waled S. Bahaj, Carmelo Gurnari, Olisaemeka D. Ogbue, Sudipto Mukherjee, Anjali Advani, James R. Cook, Heesun J. Rogers, Hetty E. Carraway, Suresh K. Balasubramanian, Valeria Visconte, Jaroslaw P. Maciejewski

**Affiliations:** 1 Department of Translational Hematology and Oncology Research, Taussig Cancer Institute, Cleveland Clinic, Cleveland, OH USA; 2 Department of Hematology and Oncology, Yale University, New Haven, CT USA; 3 Department of Biomedicine and Prevention, University of Rome Tor Vergata, Rome, Italy; 4 Department of Laboratory Medicine, Cleveland Clinic, Cleveland, OH USA; 5 Department of Hematology and Oncology, Karmanos Cancer Institute, Wayne State University, Detroit, MI USA

**Keywords:** Chemotherapy, Acute myeloid leukaemia

**TO THE EDITOR:**

Extramedullary deposits of acute myeloid leukemia (eAML), constitute an unusual presentation or complication of AML, which can occur in isolation (isolated eAML) or in the context of a fully-blown leukemic process (synchronous eAML) either at diagnosis or relapse [1–3]. This peculiar and rare variant of AML may be due to distinct biological features hypothesized to promote the homing of blasts to non-hematopoietic locations or/and be a result of specific molecular lesions, perhaps related to an invariant genotype [4, 5]. So far, systematic studies of this phenomenon have been scarce despite the advances in molecular studies and next generation sequencing over the last decade. Furthermore, while recognized in the 2022 World Health Organization (WHO) classification, guidance with regard to prognosis and treatment for this unique entity was not included in the recent European Leukemia Network (ELN) 2022 AML guidelines [6, 7]. Generally, the prognostic impact of eAML is unclear since none of the clinical and molecular prognostic tools looked at the effect of eAML on disease outcomes. Here, we took advantage of a large cohort of AML patients to investigate the clinical, molecular, and outcome features of patients with eAML. In addition, given the paucity of clinical data and randomized trials, we looked at the role of different treatment options in the management of this special entity, including isolated and synchronous eAML cases.

As a substrate for our analysis and to compare with eAML cases, we used a cohort of patients diagnosed with primary AML (pAML) and secondary AML (sAML) with a comprehensive genomic data set (*n* = 922). Patients with eAML and bone marrow (BM) involvement were considered to have synchronous eAML. Cytogenetics, molecular testing, and FISH were done on BM biopsies.

Between 2003 and 2020, we identified 91 patients (10%) with eAML, of whom 68% had synchronous and 32% isolated eAML, and with a 1:2 male:female ratio. The median age of patients with eAML (64, IQR: 57–74 years) was higher compared to our non-eAML cohort (62, IQR: 48–71 years; Table 1 and Fig. 1A). Interestingly, sAML cases were more enriched in the eAML cohort (67% vs. 40%, *p*-value < 0.001) and only 3 patients (3%) had core binding factor leukemia based on FISH from the BM. In addition, adverse risk ELN subgroup was only detected in 30% of patients with eAML vs. 52% in the non-eAML cohort (*p* < 0.001). Patients with eAML had significantly higher WBC count (7 vs. 4 × 10^9^/L, *p* = 0.002), higher hemoglobin levels (11 vs. 9 g/dL, *p* < 0.001) and higher platelet count (139 vs. 52 × 10^9^/L, *p* < 0.001) at the time of eAML diagnosis. Previous studies showed similar rates of eAML among AML patients, ranging between 9 and 14% [8–11]. Similar to our findings and in line with the favoring condition of high disease burden, patients with eAML had significantly higher WBC and peripheral blasts [8].

**Table. 1:** Baseline characteristics of our cohort.

Descriptive variable	All (%)	eAML (%)	Non-eAML (%)	*P*-value
All patients number	922	91	831	
Median (IQR) age at diagnosis, years	62 (48–72)	64 (57–74)	62 (48–71)	0.042
Gender				0.078
Male, *n*	528 (57.3)	60 (65.9)	468 (56.3)	
Female, *n*	394 (42.7)	31 (34.1)	363 (43.7)	
WHO 2016 classification				<0.001
Primary AML, *n*	460 (49.9)	27 (29.7)	433 (52.1)	
Secondary AML, *n*	395 (42.8)	61 (67.1)	334 (40.2)	
Core binding factor AML	56 (6.1)	3 (3.3)	53 (6.4)	
Median WBC at diagnosis (IQR)	8 (2–13)	7 (4–12)	4 (2–14)	0.002
Median Hb at diagnosis (IQR)	9 (8–11)	11 (9–13)	9 (8–11)	<0.001
Median platelet at diagnosis (IQR)	56 (26–112)	139 (52–289)	52 (25–101)	<0.001
Median blast at diagnosis (IQR)	29 (9–60)	3 (1–20)	32 (14–63)	<0.001
ELN 2022 risk categories				<0.001
Favorable, *n*	67 (7)	2 (2)	65 (9)	
Intermediate, *n*	396 (43)	62 (68)	334 (40)	
Adverse, *n*	459 (50)	27 (30)	432 (52)	
Cytogenetics at diagnosis				
Normal, *n*	395 (42.8)	46 (50.6)	349(42.0)	0.118
Abnormal, *n*	415 (55.8)	32 (35.2)	482 (58.0)	<0.001
Complex	188 (20.4)	12 (13.2)	176 (21.2)	0.072
Specific cytogenetics				
Deletion 5	125 (13.6)	7 (7.7)	118 (14.2)	0.085
Deletion 6	12 (1.3)	2 (2.2)	10 (1.2)	0.427
Deletion 7q	109 (11.8)	6 (6.6)	103 (12.4)	0.104
Deletion 12p	10 (1.1)	2 (2.2)	8 (1.0)	0.280
Deletion 17p	54 (5.9)	3 (3.3)	51 (6.1)	0.273
Deletion 20	25 (2.7)	3 (3.3)	22 (2.7)	0.717
Trisomy 8	97 (10.5)	12 (13.2)	85 (10.2)	0.383
-Y	37 (4.0)	5 (5.5)	32 (3.9)	0.448
*t* (8;21)	25 (2.7)	2 (2.2)	23 (2.8)	0.763
Inv 16	31 (3.4)	1 (1.1)	30 (3.6)	0.207
Number of deaths, *n*	355 (38.8)	68 (74.7)	287 (34.8)	<0.001

*eAML* extramedullary acute myeloid leukemia, *IQR* interquartile range, *WHO* world health organization, *AML* acute myeloid leukemia, *WBC* white blood cells, *Hb* hemoglobin, *ELN* European LeukemiaNet.

**Fig. 1:**
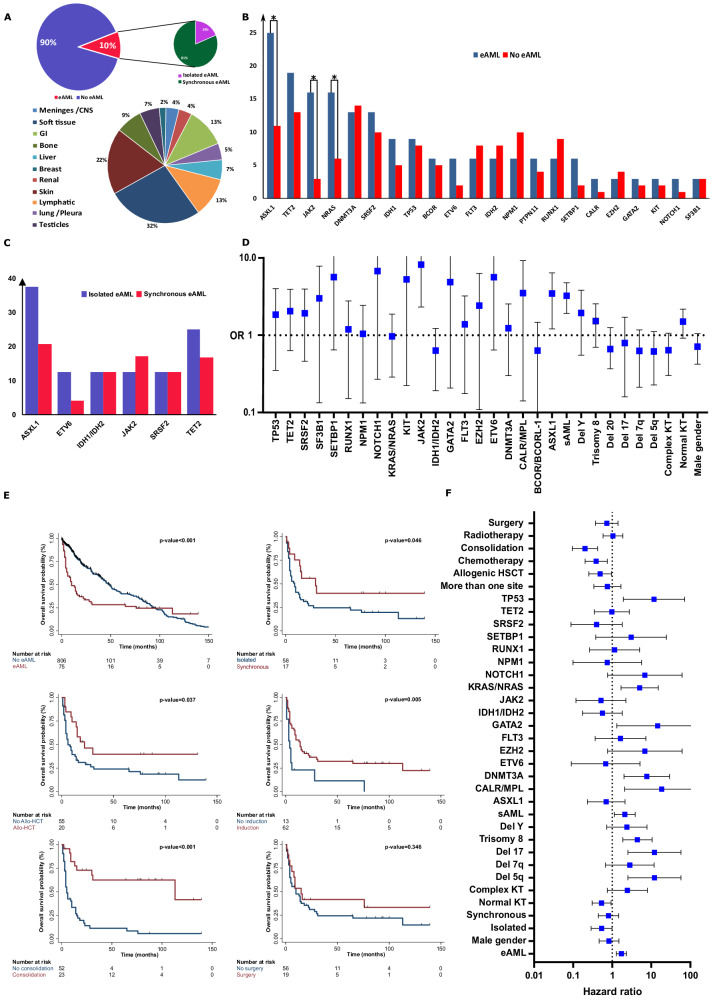
Clinical, molecular and treatment characteristics for patients with extramedullary acute myeloid leukemia. **A** Pie charts presenting the frequencies of extramedullary acute myeloid leukemia (eAML) subtypes and the most common locations for extramedullary involvement. CNS: central nervous system, GI: gastrointestinal. **B** Bar histogram presenting the most common molecular mutations in the eAML cohort compared to non-eAML patients Asterisks mark mutations with significant higher frequencies in the eAML cohort. **C** Bar histogram presenting the differences of specific mutations frequencies among patients with isolated and synchronous eAML. **D** Forest plot presenting the odd ratios (OR) for eAML based on univariable logistic regression analysis. **E** Kaplan Meier survival curve for eAML cases based on different treatment used. Allo-HCT: allogenic hematopoietic stem cell transplantation. **F** Forest plot presenting the hazard ratios for overall survival from univariable regression analysis.

Among the eAML cases, soft tissue and skin were the most common extramedullary sites (54%), followed by lymph nodes (13%, Fig. 1A). Notably, patients with isolated and synchronous eAML had similar baseline characteristics (Supplementary Table 1), besides a higher platelet count (223 vs. 118 × 10^9^/L, *p* = 0.029) in the former. Furthermore, synchronous eAML subtype had more cases involving multiple extramedullary sites (23% vs. 3%, *p*-value: 0.022).

eAML-distinctive phenotypic features were explored based on immunohistochemical (IHC) stains and flow cytometry studies. eAML neoplastic cells showed reactivity for CD43 (54% of cases), muramidase/lysozyme (53%), CD117 (39%), myeloperoxidase (35%), CD33 (32%), CD45 (30%), and CD34 (30%). Flow cytometry of the cases with synchronous eAML performed on the tumor tissue demonstrated that 90% of samples expressed surface CD13, CD33, CD45, and HLA-DR while positivity for CD38 was present in 83% of cases, and CD117 and CD34 in 60% (Supplementary Fig. 1).

In term of molecular features, *ASXL1*^*MT*^ (25%), *TET2* ^*MT*^ (19%), *JAK2* ^*MT*^ (16%), *NRAS* ^*MT*^ (16%), *DNMT3A* ^*MT*^ (13%), *SRSF2* ^*MT*^ (12%), and *IDH1* ^*MT*^ (9%) were the most common BM/peripheral blood mutations identified in the eAML cohort (Supplementary Table 2). Compared to the non-eAML cohort, patients with eAML were enriched for the following mutations: *ASXL1* ^*MT*^ (24% vs. 11%, *p* < 0.001), *JAK2* ^*MT*^ (16% vs. 3%, *p* < 0.001), and *NRAS* ^*MT*^ (16% vs. 6%, *p* = 0.041; Fig. 1B). No differences in the frequency of specific cytogenetic abnormalities were detected between eAML and non-eAML cohorts. Overall, both isolated and synchronous eAML cases had similar molecular features and cytogenetic abnormalities (Fig. 1C and Supplementary Table 3). When investigating the clinical and the molecular features associated with eAML presentation, we found that sAML (OR:2.9, 95% CI: 1.821–4.710), *JAK2*^*MT*^ (OR:6.1, 95% CI: 2.320–16.204), and *ASXL1*^*MT*^ (OR:2.8, 95% CI: 1.209–6.411) were associated with higher odds of eAML. Previous small case series reported association of eAML with specific cytogenetic abnormalities (trisomy 8 and *t*[8;21]) and genetic mutations (RAS pathway, *NPM1, IDH1/IDH2*, and *FLT3*) [2, 4, 8, 12–15].

With a median follow up time of 13.0 months (4.2–79.0), the median overall survival (OS) of patients with eAML was 10 months (95% CI: 6–16) vs. 48 months (95% CI: 43–56) in the non-eAML cohort (HR:1.7, 95% CI: 1.273–2.310). The OS at 12-months was 40% in the eAML and 82% in the non-eAML cohort. Interestingly, the OS in patients with isolated eAML was better compared to patients with synchronous eAML (30 vs. 9 months, HR:1.8; Fig. 1E, F), the OS at 12-months was 75% in the isolated eAML and 32% in the synchronous eAML cohorts.

Treatment options for all eAML cohort included chemotherapy (70%), surgical resection (24%), radiation therapy (28%), and allogenic hematopoietic stem cell transplantation ([allo-HSCT], 22%). Overall, 29 (32%) patients received multiple treatment modalities and none of the patients treated with surgery alone. The most common treatment combinations used were chemotherapy/radiation (*n* = 11, 12%), chemotherapy/surgery (*n* = 10, 11%), chemotherapy/radiation/surgery (*n* = 6, 7%), and radiation/surgery (*n* = 2, 2%). Intensive chemotherapy with 7 + 3 daunorubicin and cytarabine was used in 62% of the cases while hypomethylating agents were used in 11 patients (18%; Supplementary Table 4). High dose cytarabine for 2–3 cycles was the most common consolidation regimen (58%). Complete remission was achieved in 40/91 (44%) of the patients. Only induction chemotherapy (HR: 0.39, 95% CI: 0.201–0.750), consolidation chemotherapy (HR: 0.20, 95% CI: 0.096–0.422) and allo-HSCT (HR: 0.49, 95% CI: 0.252–0.959) were associated with favorable OS. *TP53* ^*MT*^, *KRAS/NRAS* ^*MT*^, *DNMT3A* ^*MT*^, del(5q), trisomy 8, and del17p were associated with poor OS among patients with eAML (Fig. 1F). Using multivariable Cox proportional hazard model (adjusted for significant variable in univariate analysis), consolidation chemotherapy (HR: 0.23, 95% CI: 0.098–0.543) was the only treatment measure associated with significant OS benefit. Del(5q) (HR:13.9, 95% CI: 1.556–124.343) and trisomy 8 (HR:3.0, 95% CI: 1.159–7.624) were both associated with worse outcomes.

In our large cohort of AML patients, 10% of the patients diagnosed with eAML. Patients with eAML exhibited a higher circulating disease burden at the time of the diagnosis, consistent with the loss of BM homing of leukemic blasts and systemic spreading capacity. *ASXL1* ^*MT*^, *JAK2* ^*MT*^, and *NRAS* ^*MT*^ were significantly enriched in the eAML cohort. Notably, patients with eAML had worse OS compared to the other AML subtypes, especially cases with synchronous eAML. Since patients with eAML are usually excluded from clinical trials, no standard treatment protocols have been established for the management of this unique population. Our findings showed that patients who were able to receive cytarabine-based chemotherapy had better outcomes as opposed to other treatment options, including surgical resection, radiation therapy, and even allo-HSCT.

In our cohort, cases with eAML were enriched with the RAS pathway, *ASXL1*, and *JAK2* mutations. No specific cytogenetic abnormalities were associated with eAML cases or different eAML subtypes. However, the presence of trisomy 8 and del(5q) was associated with worse OS as shown in our cohort.

Although some reports found no impact on OS [16, 17], eAML was associated with worse survival in our cohort (10 vs. 48 months). eAML was reported to be an independent risk factor in a large cohort of 225 cases of eAML (14 vs. 26.2 months) [8]. Given the rarity of the condition, no specific guidelines have been established for treating this specific entity of AML. Treatment options reported in the literature include intensive chemotherapy with 7 + 3 induction followed by consolidation, hypomethylating agents and venetoclax, *FLT3* and *IDH1/IDH2* targeting (when amenable), radiation therapy and surgical interventions [1, 12, 18–21]. Allo-HSCT can provide survival benefits in patients who achieve complete remission [8, 20]. None of the chemotherapy regimens, including targeted therapeutic options, have been reported to provide a better response or OS rates in a large number of patients. Targeted therapy with gilteritinib has shown to be effective as a bridging therapy for allo-HCT in a few cases with *FLT3*-mutant eAML [12, 15, 22]. Allo-HSCT did not improve OS in our cohort. This can be attributed to the small number of eAML who underwent transplantation (*n* = 20, 22%). The limited efficacy of allo-HSCT in cases with eAML may be related to extensive extramedullary involvement, making complete remission before allo-HSCT harder to achieve, with higher post-transplant relapse rates (20% relapse among cases with eAML were treated with allo-HSCT). However, only two patients with eAML treated with hypomethylating agents/venetoclax and these two patients did not have allo-HSCT.

In conclusion, eAML represents an uncommon manifestation of AML with a diverse molecular profile and worse clinical outcomes compared to other types of AML. Using standard genomics and clinical and pathomorphological parameters, we were unable to identify a distinct configuration of features separating eAML from other forms of AML. It is possible that extramedullary conglomerations of leukemic blasts represent a separate subclone with biologic features that differ from circulating leukemia. Treatment of eAML may include chemotherapy with the possible addition of targeted therapy in particular cases. The role of allo-HCT in eAML is controversial as in our cohort only a few patients were able to undergo such procedure. Further studies including larger number of patients with eAML treated with allo-HSCT are warranted. Finally, prospective studies are difficult to envision in such a setting, given the rarity of the condition. Therefore, including patients with eAML in clinical trials should be encouraged.

### Supplementary information

Supplementary Material

## Data Availability

Requests for additional information not provided in the main text or supplementary material should be sent to the corresponding author.
