# Supplementary material for: Clinical and molecular characteristics of extramedullary acute myeloid leukemias
Source: Leukemia. 2024 Jul 18;38(9):2032–6. doi: 10.1038/s41375-024-02337-0 (PMC11347362; doi:10.1038/s41375-024-02337-0)
Supplement: Supplementary file 1 — Supplementary Material [file 41375_2024_2337_MOESM1_ESM.docx]

**SUPPLEMENTAL MATERIAL**

**Clinical and Molecular Characteristics of Extramedullary Acute Myeloid Leukemias**

Tariq Kewan^1,2^, Waled S. Bahaj^1^, Carmelo Gurnari^1,3^, Olisaemeka D. Ogbue^1^, Sudipto Mukherjee^1^, Anjali Advani^1^, James R. Cook^4^, Heesun J. Rogers^4^, Hetty E. Carraway^1^, [Suresh K. Balasubramanian](https://pubmed.ncbi.nlm.nih.gov/?term=Balasubramanian+SK&cauthor_id=31648302)^5^, Valeria Visconte^1^, Jaroslaw P. Maciejewski^1*^

^1^Department of Translational Hematology and Oncology Research, Taussig Cancer Institute, Cleveland Clinic, Cleveland, OH.

^2^Department of Hematology and Oncology, Yale university, New Haven, CT.

^3^Department of Biomedicine and Prevention, Ph.D. in Immunology, Molecular Medicine and Applied Biotechnology, University of Rome Tor Vergata, Rome, Italy.

^4^Department of Pathology, Cleveland Clinic, Cleveland, OH.

^5^Department of Hematology and Oncology, Karmanos Cancer Institute, Wayne State University, Detroit, MI

**Content:**

**A) Supplementary tables**

- **Supplementary Table-1.** Baseline characteristics of isolated and synchronous extramedullary AML cases.
- **Supplementary Table-2.** Molecular characteristics of all acute myeloid leukemia cases including cases with extramedullary acute myeloid leukemia (eAML).
- **Supplementary Table-3.** Molecular characteristics of isolated and
  synchronous extramedullary acute myeloid leukemia cases.
- **Supplementary Table-4**. Induction and consolidation treatment used in our cohort.

**B) Supplementary figures**

- **Supplementary Figure 1.** Flow cytometry features of extramedullary acute myeloid leukemia (eAML) cases.

**Supplementary Table-1.** Baseline characteristics of isolated and synchronous extramedullary acute myeloid leukemia cases.

| Descriptive variable | All (%) | Isolated eAML (%) | Synchronous eAML (%) | P-value |
| --- | --- | --- | --- | --- |
| All patients number | 91 | 29 | 62 |  |
| Median (IQR) age at diagnosis, years | 64 (57-74) | 64 (55-74) | 64 (59-74) | 0.387 |
| Gender |  |  |  | 0.314 |
| Male | 60 (65.9) | 17 (59) | 43 (69) |  |
| Female | 31 (34.1) | 12 (41) | 19 (31) |  |
| Concurrent AML | 29 | 11 (38) | 21 (34) | 0.705 |
| Median WBC at diagnosis | 7 (4-12) | 6 (5-8) | 7 (4-16) | 0.327 |
| Median Hb at diagnosis | 11 (9-13) | 11 (10-13) | 11 (9-13) | 0.297 |
| Median platelet at diagnosis | 139 (52-289) | 223 (90-300) | 118 (48-217) | 0.029 |
| Median blast at diagnosis | 3 (1-20) | 2 (1-32) | 4 (1-15) | 0.889 |
| Cytogenetics at diagnosis |  |  |  |  |
| Normal | 46 (50.6) | 15 (52) | 31 (50) | 0.878 |
| Abnormal | 32 (35.2) | 12 (41) | 20 (32) | 0.396 |
| Complex | 12 (13.2) | 2 (7) | 10 (16) | 0.225 |
| Specific cytogenetics |  |  |  |  |
| Deletion 5 | 7 (7.7) | 1 (3) | 6 (10) | 0.299 |
| Deletion 6 | 2 (2.2) | 0 (0) | 2 (3) | 0.328 |
| Deletion 7q | 6 (6.6) | 0 (0) | 6 (10) | 0.083 |
| Deletion 12p | 2 (2.2) | 1 (3) | 1 (2) | 0.578 |
| Deletion 17p | 3 (3.3) | 0 (0) | 3 (5) | 0.228 |
| Deletion 20 | 3 (3.3) | 0 (0) | 3 (5) | 0.228 |
| Trisomy 8 | 12 (13.2) | 1 (3) | 11 (18) | 0.060 |
| -Y | 5 (5.5) | 2 (7) | 3 (5) | 0.688 |
| MS location |  |  |  |  |
| Skin | 20 (22.0) | 7 (24) | 13 (21) | 0.734 |
| Soft tissue | 29 (31.9) | 11 (38) | 18 (29) | 0.396 |
| Lymph nodes | 12 (13.2) | 3 (10) | 9 (15) | 0.584 |
| GI tract | 12 (13.2) | 3 (10) | 9 (15) | 0.584 |
| Liver | 6 (6.6) | 3 (10) | 3 (5) | 0.324 |
| Bone | 8 (8.8) | 2 (7) | 6 (10) | 0.662 |
| Lung/Pleura | 5 (5.5) | 0 (0) | 5 (8) | 0.116 |
| Testicles | 6 (6.6) | 0 (0) | 6 (10) | 0.083 |
| Breast | 2 (2.2) | 0 (0) | 2 (3) | 0.328 |
| Meninges | 4 (4.4) | 0 (0) | 4 (6) | 0.162 |
| Renal | 4 (4.4) | 1 (3) | 3 (5) | 0.763 |
| More than one site | 15 (16.5) | 1 (3) | 14 (23) | 0.022 |
| Treatments |  |  |  |  |
| Induction chemotherapy | 64 (70.3) | 24 (83) | 40 (65) | 0.076 |
| Consolidation chemotherapy | 24 (26.4) | 16 (55) | 8 (13) | 0.000 |
| Surgery | 22 (24.2) | 7 (24) | 15 (24) | 0.995 |
| Radiotherapy | 25 (27.5) | 7 (24) | 18 (29) | 0.626 |
| Allogeneic BMT | 20 (22.0) | 5 (17) | 15 (24) | 0.456 |
| Outcomes |  |  |  |  |
| Dead | 68 (74.7) | 17 (59) | 51 (82) | 0.016 |
| Relapse after remission | 13 (14.3) | 5 (17) | 8 (13) | 0.582 |

**Supplementary Table-2.** Molecular characteristics of all acute myeloid leukemia cases including cases with extramedullary acute myeloid leukemia.

| Mutations | All (%) | eAML (%) | No eAML (%) | P-value |
| --- | --- | --- | --- | --- |
| APC | 11 | 0 | 11 (2) | 0.424 |
| ASXL1 | 85 | 8 (25) | 77 (11) | 0.012 |
| BCOR | 37 | 2 (6) | 35 (5) | 0.772 |
| BCORL1 | 20 | 0 | 20 (3) | 0.338 |
| CALR | 4 | 1 (3) | 3 (1) | 0.109 |
| CBL | 22 | 0 | 22 (3) | 0.316 |
| CEBPA | 6 | 0 | 6 (1) | 0.577 |
| CUX1 | 13 | 0 | 13 (2) | 0.409 |
| DDX41 | 10 | 0 | 10 (2) | 0.470 |
| DNMT3A | 106 | 4 (13) | 102 (14) | 0.800 |
| ETV6 | 18 | 2 (6) | 16 (2) | 0.145 |
| EZH2 | 28 | 1 (3) | 27 (4) | 0.856 |
| FLT3 | 62 | 2 (6) | 59 (8) | 0.699 |
| GATA2 | 15 | 1 (3) | 14 (2) | 0.640 |
| IDH1 | 37 | 3 (9) | 34 (5) | 0.257 |
| IDH2 | 57 | 2 (6) | 55 (8) | 0.771 |
| JAK2 | 28 | 6 (16) | 22 (3) | 0.000 |
| KDM6A | 16 | 0 | 16 (3) | 0.359 |
| KIT | 14 | 1(3) | 13 (2) | 0.586 |
| KRAS | 17 | 0 | 17 (2) | 0.380 |
| MPL | 5 | 0 | 5 (1) | 0.636 |
| NF1 | 15 | 0 | 15 (4) | 0.270 |
| NOTCH1 | 10 | 1 (3) | 9 (1) | 0.452 |
| NPM1 | 66 | 2 (6) | 64 (10) | 0.441 |
| NRAS | 51 | 5 (16) | 46 (6) | 0.041 |
| PHF6 | 21 | 0 | 21 (3) | 0.327 |
| PTPN11 | 28 | 2 (6) | 26 (4) | 0.439 |
| RAD21 | 12 | 0 | 12 (2) | 0.428 |
| RUNX1 | 69 | 2 (6) | 67 (9) | 0.558 |
| SETBP1 | 18 | 2 (6) | 16 (2) | 0.145 |
| SF3B1 | 23 | 1 (3) | 22 (3) | 0.982 |
| SMC3 | 9 | 0 | 9 (1) | 0.493 |
| SRSF2 | 73 | 4 (13) | 69 (10) | 0.584 |
| STAG2 | 41 | 0 | 41 (7) | 0.134 |
| SUZ12 | 7 | 0 | 7 (2) | 0.480 |
| TET2 | 99 | 6 (19) | 93 (13) | 0.324 |
| TP53 | 61 | 3 (9) | 58 (8) | 0.783 |
| U2AF1 | 45 | 0 | 45 (6) | 0.145 |
| WT1 | 20 | 0 | 20 (3) | 0.340 |
| ZRSR2 | 17 | 0 | 17 (2) | 0.380 |

**Supplementary Table-3.** Molecular characteristics of isolated and
synchronous extramedullary acute myeloid leukemia cases.

| Mutations | All (%) | Isolated eAML (%) | Synchronous eAML (%) | P-value |
| --- | --- | --- | --- | --- |
| ASXL1 | 8 (25) | 4 (29) | 4 (22) | 0.681 |
| BCOR | 2 (6) | 1 (7) | 1 (6) | 0.854 |
| CALR | 1 (3) | 0 | 1 (6) | 0.370 |
| DNMT3A | 4 (13) | 1 (7) | 3 (17) | 0.419 |
| ETV6 | 2 (6) | 2 (14) | 0 | 0.098 |
| EZH2 | 1 (3) | 1 (7) | 0 | 0.249 |
| FLT3 | 2 (6) | 0 | 2 (11) | 0.198 |
| GATA2 | 1 (3) | 0 | 1 (6) | 0.370 |
| IDH1 | 3 (9) | 0 | 3 (16) | 0.119 |
| IDH2 | 2 (6) | 1 (7) | 1 (6) | 0.854 |
| JAK2 | 6 (16) | 1 (7) | 5 (22) | 0.243 |
| KIT | 1(3) | 0 | 1 (6) | 0.370 |
| NOTCH1 | 1 (3) | 1 (7) | 0 | 0.249 |
| NPM1 | 2 (6) | 1 (7) | 1 (6) | 0.854 |
| NRAS | 5 (16) | 1 (7) | 4 (22) | 0.244 |
| PTPN11 | 2 (6) | 1 (7) | 1 (6) | 0.854 |
| RUNX1 | 2 (6) | 1 (7) | 1 (6) | 0.854 |
| SETBP1 | 2 (6) | 0 | 2 (11) | 0.198 |
| SF3B1 | 1 (3) | 0 | 1 (6) | 0.370 |
| SRSF2 | 4 (13) | 1 (7) | 3 (17) | 0.419 |
| TET2 | 6 (19) | 2 (14) | 4 (22) | 0.568 |
| TP53 | 3 (9) | 1 (7) | 2 (11) | 0.702 |

**Supplementary Table-4**. Induction and consolidation
treatment used in our cohort.

| Chemotherapy | N (%) |
| --- | --- |
| Induction | **63** |
| Daunorubicin + cytarabine (7+3) | 39 (62) |
| Low dose cytarabine (LDAC) | 7 (11) |
| clofarabine +cytarabine | 3 (5) |
| HMA | 8 (13) |
| Azacitidine + Venetoclax | 2 (3) |
| Azacitidine +Gilteritinib | 1 (2) |
| MEC | 3 (5) |
| Consolidation and second line treatment | **24** |
| HiDAC | 14 (58) |
| Daunorubicin + cytarabine (5+2) | 4 (17) |
| Daunorubicin + cytarabine (7+3) | 2 (8) |
| Intermediate dose cytarabine | 1 (4) |
| MEC | 1 (4) |
| MEC+bortozimib | 1 (4) |
| clofarabine +cytarabine | 1 (4) |

**Supplementary Figure-1**

**Supplementary Figure-1.** Bar graph presenting the percentage of extramedullary acute myeloid leukemia (eAML) samples (n=32) expressing different surface markers (x-axis).
